# Parallel Declines in Human Papillomavirus 6/11–Related Disease Incidences: A National Study of Anogenital Warts and Recurrent Respiratory Papillomatosis in Norway

**DOI:** 10.1093/ofid/ofag456

**Published:** 2026-07-29

**Authors:** Madleen Orumaa, Ståle Nygård, Joseph Tota, Parag Mahale, Thea Eline Hetland Falkenthal

**Affiliations:** Department of Research, Cancer Registry of Norway, Norwegian Institute of Public Health, Oslo, Norway; Department of Research, Cancer Registry of Norway, Norwegian Institute of Public Health, Oslo, Norway; Department of Epidemiology, Biostatistics and Research Decision Sciences, MRL, Merck & Co., Inc., Rahway, New Jersey, USA; Department of Epidemiology, Biostatistics and Research Decision Sciences, MRL, Merck & Co., Inc., Rahway, New Jersey, USA; Department of Research, Cancer Registry of Norway, Norwegian Institute of Public Health, Oslo, Norway

**Keywords:** quadrivalent HPV vaccine, AoRRP, JoRRP, RRP, AGW

## Abstract

Anogenital warts and recurrent respiratory papillomatosis are conditions caused by human papillomavirus (HPV) types 6/11 that can be prevented by vaccination. We analyzed Norwegian national registry data (2010–2023) to assess the impact of the 4-valent HPV vaccine introduced in 2009. Anogenital warts incidence among females aged 15–29 decreased significantly from 1120.0 to 280.6 per 100 000 (annual percentage change: −10.3%), paralleling a sharp decline in recurrent respiratory papillomatosis from 3.7 to 0.6 per 100 000 (annual percentage change: −11.3%). These results strongly support vaccination impact in reducing the burden of HPV 6/11-related diseases, suggesting successful interruption of transmission pathways and substantial population-level protection.

## BACKGROUND

Anogenital warts (AGW) are predominantly caused by human papillomavirus (HPV) types 6/11, with prevalence peaking in young adults aged 15 to 24 years due to sexual activity and susceptibility [1]. Although treatable with cryotherapy or ointments, such as podophyllotoxin, the condition remains clinically and psychosocially burdensome because of high recurrence rates [1].

Recurrent respiratory papillomatosis (RRP) is a disease also caused by HPV types 6/11, characterized by typically benign tumors that grow in the respiratory tract, most often the larynx [2]. The disease manifests in two forms: a more aggressive juvenile-onset (JoRRP) version, which results from vertical (mother-to-child) HPV transmission, and usually diagnosed at ages 0 to 14 years [3]; and adult-onset (AoRRP), which is diagnosed after 15 years of age and is likely sexually acquired. Because no cure currently exists, management relies on repeated surgical interventions to maintain airway patency, imposing a significant physical and financial burden on patients [4].

Before the introduction of HPV vaccines, the RRP incidences in southeastern Norway—as in many other developed countries—were stable at 0.17 and 0.54 per 100 000 for JoRRP and AoRRP, respectively [5]. Since the introduction of prophylactic 4-valent and 9-valent HPV vaccines, which protect against the causative HPV 6/11 types, there has been expected declines in the incidence of both AGWs and RRP in countries with high-coverage immunization programs, such as Australia [6, 7] and the United States [8].

**Figure 1.**
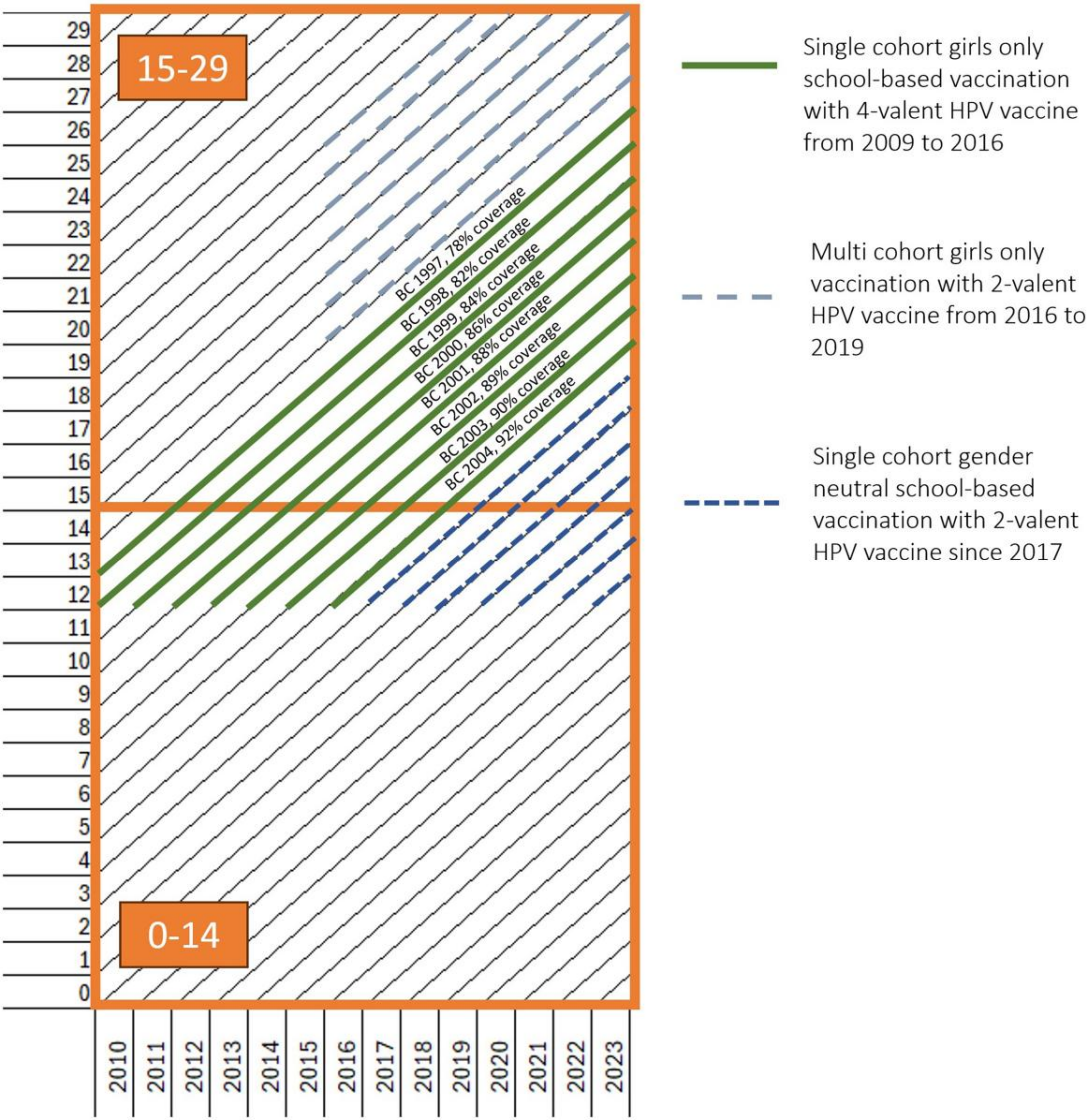
Lexis diagram of the Norwegian HPV vaccination program by birth cohort and age (2010–2023). The diagonal bands trace individual birth cohorts as they age over calendar time, illustrating exactly when vaccinated cohorts enter the high-risk 15- to 29-year age bracket. Green solid lines represent cohorts receiving the 4-valent vaccine (with coverage rates indicated). Dashed lines represent cohorts vaccinated with the 2-valent vaccine in the national school-based program. Abbreviations: BC, birth cohort; HPV, human papillomavirus.

However, Norway's evaluation provides a unique epidemiological context that differs significantly from Australia or the United States. While Australia rapidly implemented a high-coverage, multicohort, gender-neutral program and the United States relied on opportunistic mixed-market delivery, Norway initially introduced a strictly female-only, single-cohort school-based program targeted at 12-year-old girls in 2009 (Figure 1).

In 2016, the program transitioned to the 2-valent vaccine (which lacks protection against HPV 6/11), initially offering it to multicohort of girls. From 2018, the program became gender-neutral, extending the 2-valent vaccination to include boys. Because the cohorts with the highest historical incidence of AGW were already covered by the 4-valent HPV vaccine during their peak risk windows, a resurgence of AGW has not yet been observed despite the shift to the 2-valent vaccine. Despite the 4-valent single cohort vaccination, excellent impact against AGW [9] and on cervical precancerous lesions has been documented [10].

This study aims to investigate the epidemiological trends in the incidences of AGW among females aged 15 to 29 years and of RRP among males and females aged 0 to 29 years from 2010 to 2023, following the introduction of the 4-valent HPV vaccine.

## MATERIAL AND METHODS

### Study Population

We retrieved population statistics from the Statistics Norway open resources, covering 2010–2023 by single year of age [11].

### Outcomes

To estimate trends in AGW incidence, we used publicly available data from the Norwegian Prescribed Drug Registry [12]. Prescriptions for podophyllotoxin (ATC code D06BB04), the predominant pharmacological treatment for AGW in Norway, served as a proxy for disease occurrence. This proxy method is validated by our group's previous individual-level linkages between the Norwegian Prescribed Drug Registry and the Norwegian Patient Registry, which demonstrated that only 13% of AGW patients had a unique record appearing in the Patient Registry alone (representing surgical or specialized care) without a matching prescription. This confirms that podophyllotoxin data captures the clear majority (87%) of the clinically managed burden. In a minority of AGW cases, imiquimod is prescribed. However, because this medication is also prescribed for other conditions, most notably small superficial basal-cell carcinomas, prescription for imiquimod was not used to define AGW cases to avoid losing specificity. We extracted the annual number of treated females aged 15 to 29 years for the period 2010–2023.

Cases of RRP were identified in the Norwegian Patient Registry using the International Classification of Diseases, 10th Revision (ICD-10) code D14.1 (benign neoplasm of the larynx). Procedures related to the management of RRP were identified using the Norwegian Clinical Procedure Coding System. Surgical interventions were extracted using codes DQB10 (endoscopic extirpation), UDQ22/DUQ22 (microlaryngoscopy), and UDQ25/DUQ25 (microlaryngoscopy with biopsy). For each individual, we extracted data on the year of the first D14.1 diagnosis, sex, and age at diagnosis. To ensure accuracy, duplicate records were removed, and only primary diagnoses were considered. Individuals were included if they had a first recorded diagnosis of D14.1 during the study period and at least 1 documented surgical treatment at any time. To avoid prevalent cases, we excluded 2 previous years (2008–2009) of registered cases.

Vaccination coverage rates for 4- and 9-valent HPV vaccines among females aged 15 to 29 years (vaccinated before age 15 years) were derived from the Norwegian Immunisation Registry and the Norwegian Prescribed Drug Registry. The former contains all vaccination through the childhood program, whereas the latter captures opportunistic vaccination, which since 2015 have mostly been with the 9-valent vaccine.

### Statistics

To characterize the impact of direct HPV vaccine receipt, we calculated AGW and RRP incidence rates together with 95% confidence intervals (CI) using Poisson regression for females aged 15–29 per 100 000 persons in 2-year intervals. For males and the 0–14 age group, RRP incidence rates were reported for aggregated periods (2010–2014, 2015–2019, 2020–2023) to provide robust estimates of background trends and indirect effects.

To assess temporal trends, we calculated annual percentage changes (APC) with 95% CIs from annual data using a Poisson model with the log of person-years as an offset. Although these formal trend models used true annual data to maximize statistical precision, visual presentations and descriptive text comparisons relied on the grouped intervals. This data aggregation was required to satisfy national data privacy mandates because individual yearly cell counts frequently fell below the cell suppression threshold of 5 cases, publishing raw annual frequencies would conflict with strict anonymity guidelines.

All tests were 2-sided, and a *P* < .05 was considered statistically significant. Statistical software Stata 19.5 was used for analysis.

## RESULTS

A total of 47 043 incident AGW cases (defined by new prescriptions) were identified among females aged 15 to 29 years from 2010–2023. During the same period, 242 RRP cases with at least 1 surgical treatment were diagnosed in 127 (52.5%) females and 115 (47.5%) males.

**Figure 2.**
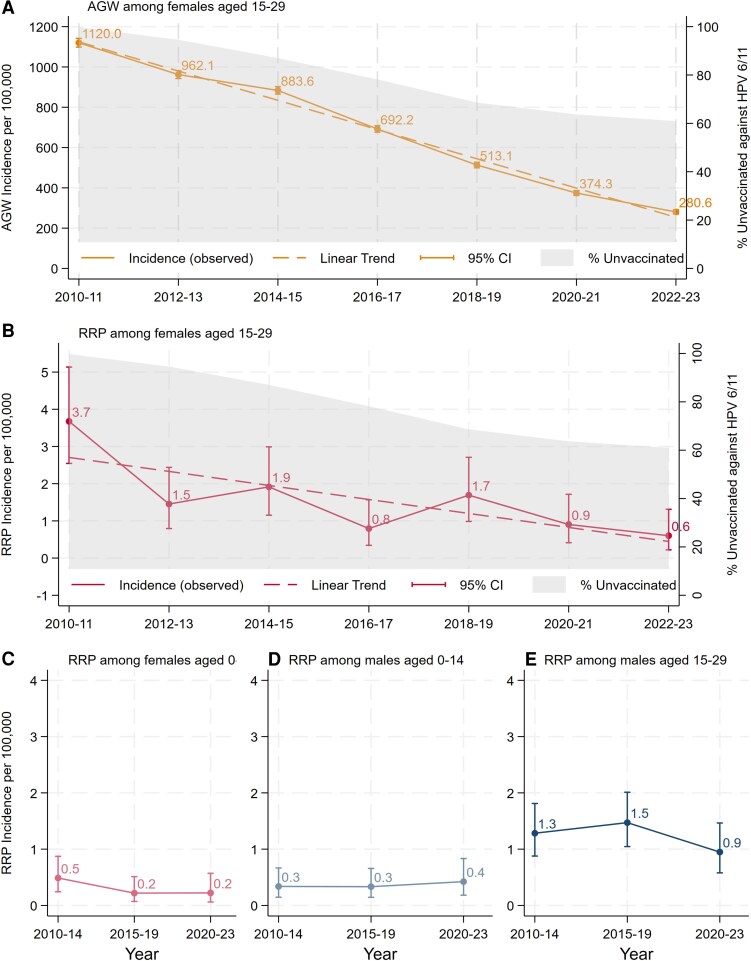
Trends in the incidence of anogenital warts (AGW) and recurrent respiratory papillomatosis (RRP) alongside increasing human papillomavirus (HPV) vaccination coverage (2010–2023). (*A*) Observed annual incidence, linear trend, and 95% confidence intervals (CIs) of AGW per 100 000 females aged 15–29 years, plotted against the percentage of the cohort unvaccinated against HPV 6/11. (*B*) Observed annual incidence, linear trend, and 95% CIs of RRP per 100 000 females aged 15–29 years, plotted against the percentage of the cohort unvaccinated against HPV 6/11. (*C–E*) RRP incidence per 100 000% and 95% CIs among females aged 0–14 years (*C*), males aged 0–14 years (*D*), and males aged 15–29 years (*E*). All confidence intervals are calculated using the exact Poisson method. For (*A*) 95% CIs are narrow and might be difficult to note.

Among females aged 15 to 29 years, the AGW incidence rates declined from 1120.0 to 280.6 per 100 000 (APC: −10.3%; 95% CI: −10.7% to −10.2%) (Figure 2*A*). Similarly, RRP incidence rate decreased significantly from 3.7 per 100 000 in 2010–2011 to 0.6 in 2022–2023, with an APC of −11.3% (95% CI: −15.7% to −6.8%) (Figure 2*B*). These reductions occurred concomitantly with a substantial decrease in the unvaccinated fraction of the cohort (from 99% to 60%) (Figure 2*A* and 2*B*). The RRP incidence rate for females aged 0 to 14 years decreased from 0.5 (in 2010–2014) to 0.2 (in 2020–2023) per 100 000 females, corresponding to an APC of −11.9% (95% CI: −22.3%; −1.5%) (Figure 2*C*).

Among males aged 15 to 29 years, the RRP incidence rate decreased from 1.3 to 0.9 per 100 000 over the same periods, representing a nonsignificant APC of −3.3% (95% CI: −8.3% to 1.7%). For males aged 0 to 14 years, the incidence rate remained stable (0.3 in 2010–2014 to 0.4 in 2020–2023; APC: 1.8% [95% CI: −8.3%; 12.0%]) (Figure 2*C*–2*E*).

## DISCUSSION

To our knowledge, this is the first study to report national RRP incidence trends in Norway. Together with AGW incidence, we demonstrated a strong decrease in HPV6/11-related disease incidence among females aged 15 to 29 years following the inclusion of the 4-valent HPV vaccine in the childhood vaccination program, suggesting a plausible direct and indirect vaccine effect.

AoRRP is predominantly acquired through sexual transmission, with oral-genital contact serving as the primary route of viral entry for HPV types 6/11 [1]. Given that these specific genotypes are responsible for more than 90% of AGW and the vast majority of RRP lesions, AGW incidence serves as a reliable clinical proxy for the circulating burden of infectious HPV 6/11. The effectiveness of the 4-valent HPV vaccine in the Norwegian population is well established, with a significant decrease in AGW [9] and cervical lesions [10] noted among vaccinated cohorts. We hypothesize that this reduction in the genital viral reservoir leading to a reduced burden of AGW has also altered the transmission dynamics required for AoRRP. By minimizing genital HPV 6/11 prevalence, the probability of oral acquisition during sexual activity is reduced.

Vaccine coverage in target group is a crucial factor influencing disease incidence. Although only 8 birth cohorts (1997–2004) of girls in Norway received the vaccine covering HPV 6/11, the high vaccine coverage (80%–90%) in these cohorts has produced remarkable protection (Figure 1). The declines in incidences in women aged 15 to 29 years were 75% (from 1120 in 2010–2011 to 228 in 2022–2023 per 100 000) for AGWs and 84% (from 3.7 to 0.6 per 100 000) for RRP, which was much higher than the decrease in proportion of HPV 6/11-unvaccinated women in this age group (40%, from 100% in 2010–2011 to 60% in 2022–2023). This indicates a herd protection effect, which is consistent with previous findings for AGW [13], and in line with the decline in RRP incidence among largely unvaccinated males aged 15 to 29 years, although not statistically significant in our APC analysis.

Crucially, JoRRP incidence epidemiologically reflects the vaccination status of the mother rather than the child, given that exposure occurs via vertical transmission during childbirth. This maternal-dependent mechanism explains why we observe a stable RRP trend among young males aged 0 to 14 years. Throughout our 2010–2023 study window, the vast majority of children aged 0 to 14 years was born to older maternal cohorts who fell outside the vaccine rollout and were entirely unprotected. The first wave of vaccinated girls only reached peak childbearing ages at the absolute tail end of our data window, meaning maternal herd protection has not yet had sufficient time to manifest in the pediatric male population. Although not significant, there was a decline observed among pediatric females aged 0 to 14 years, which might be driven by direct vaccine protection rather than maternal immunity. Nevertheless, it is critical to emphasize that the absolute number of JoRRP cases within these pediatric cohorts is very small, implying that the analysis lacks the statistical power required to establish definitive or robust conclusions. These early sex-specific trends are highly susceptible to minor statistical fluctuations and must be interpreted with a high degree of caution.

This study provides real-world evidence supporting the effectiveness of Norway's national HPV vaccination program including the 4-valent HPV vaccine from 2009 to 2016. It supports the value of vaccines (4-valent or 9-valent) that includes genotypes 6 and 11, which are highly effective in preventing certain cancers and AGW, but may also prevent RRP, which imposes a substantial burden on patients, their families, and healthcare systems due to their chronic nature and need for repeated interventions. Considering the vertical mode of acquisition of causal HPV infection in JoRRP cases, immunization of women against HPV 6/11 likely also protects their unborn children. By reducing maternal HPV 6/11 infection and associated AGW—the principal risk factor for JoRRP—vaccination represents an important potential opportunity for mitigating the burden of this life-threatening condition in children [3, 14].

This study benefits from the use of comprehensive, national population-based registry data on RRP incidence, minimizing the risk of selection bias and providing a complete epidemiological picture. Furthermore, by defining cases based on confirmed treatment codes rather than diagnostic codes alone, we likely achieved a high degree of specificity. However, limitations exist. First, the ecological design prevents linking individual vaccination status to cases, creating a risk of ecological fallacy. Second, relying on treatment records may miss mild, untreated AGW and RRP cases. Third, the use of proxy measures for case identification introduces diagnostic uncertainty. For AGW, we relied on podophyllotoxin prescriptions without linkage to ICD-10 code A63.0; lack of individual-level diagnostic validation may overestimate incidence as prevalent cases. Similarly, identifying RRP cases using ICD-10 code D14.1 (benign neoplasm of larynx) may lead to misclassification, as this code could inadvertently capture other benign laryngeal lesions. Furthermore, although imiquimod is another available treatment, its usage among young people in Norway is minimal and likely does not skew trends. Finally, because our analysis relies on indirect clinical proxies and administrative drug registry, the possibility of unmeasured historical confounders, such as shifting healthcare utilization patterns, changes in clinical diagnostic practices, or evolving physician prescribing preferences, represents a major limitation of this analysis and cannot be completely ruled out.
